# Supplementary material for: Content-rich biological network constructed by mining PubMed abstracts
Source: BMC Bioinformatics. 2004 Oct 8;5:147. doi: 10.1186/1471-2105-5-147 (PMC528731; doi:10.1186/1471-2105-5-147)
Supplement: Additional File 5 — The original Chilibot query results of the term "long-term potentiation (LTP)" and 22 other terms, limiting the latest references analyzed to the years 1990, 1995, 2000, and 2004. [file 1471-2105-5-147-S5.bz2 › chilibotAdditionalFile5/ltp2004/html/AMPA_ZIF268.html]

 


 **AMPA** and **ZIF268** 
  
Found 1 abstracts in PubMed,  **1 abstracts were retrieved and analyzed**.  


---

 Search Google  |
 PDF files only 
|  EDU domain only 

---

- J Neurochem, 2002   The acute hippocampal slice preparation is a convenient, in vitro model widely used to study the biological basis of synaptic plasticity.
  Although slices may preserve their electrophysiological properties for several hours, profound molecular changes in response to the injury caused by the slicing procedure are likely to occur.
  To determine the magnitude and duration of these changes we examined the post slicing expression kinetics of three classes of genes known to be implicated in long term synaptic plasticity glutamate **AMPA** receptors , transcription factors and neurotrophins.
  Slicing resulted in a striking loss of GluR1 and GluR3, but not of GluR2 proteins suggesting that rapid changes in the composition of major neurotransmitter receptors may occur.
  Slicing caused a significant induction of the transcription factors c fos, **zif268**, CCAAT enhancer binding protein beta and delta mRNAs and of the neurotrophin brain derived neurothophic factor mRNA.
  In contrast, there was no augmentation, and sometimes a decline, in the levels of the corresponding proteins.
  These data reveal that significant discrepancies exist between the slice preparation and the intact hippocampus in terms of the metabolism of molecular components known to be involved in synaptic plasticity.
